# OVERWEIGHT AND ASSOCIATED FACTORS IN CHILDREN AND ADOLESCENTS WITH PHENYLKETONURIA: A SYSTEMATIC REVIEW

**DOI:** 10.1590/1984-0462/2020/38/2018201

**Published:** 2020-03-09

**Authors:** Berilany dos Santos Sena, Maria Izabel Siqueira de Andrade, Ana Paula Ferreira da Silva, Keila Fernandes Dourado, Andressa Laís Ferreira Silva

**Affiliations:** a Universidade Federal de Pernambuco, Vitória de Santo Antão, PE, Brazil.; b Hospital Barão de Lucena, Recife, PE, Brazil.

**Keywords:** Phenylketonurias, Overweight, Child, Adolescent, Review, Fenilcetonúrias, Sobrepeso, Criança, Adolescente, Revisão

## Abstract

**Objective:**

To verify the occurrence of overweight in children and adolescents with phenylketonuria and to identify possible causal factors.

**Data sources:**

A systematic review was performed in the SciELO, PubMed and VHL databases using the descriptors “Phenylketonurias”, “Overweight”, “Child” and “Adolescent”. Original articles conducted with children and adolescents, published between 2008 and 2018 in Portuguese, English or Spanish languages were included.

**Data synthesis:**

A total of 16 articles were identified and, after screening procedures, 6 studies were selected for the review. Overweight in children and adolescents with phenylketonuria was a frequent occurence in the studies included in this review, ranging from 7.8 to 32.6%. The female sex was the most affected by the nutritional disorder. Furthermore, a high caloric intake combined with a lack of stimuli to practice physical activities were main factors associated with the excessive weight in the population of interest.

**Conclusions:**

Excess weight can be considered a common outcome in children and adolescents with phenylketonuria. It is mainly caused by inadequate food consumption and sedentary lifestyle. The importance of early identification of nutritional disturbances in children and adolescents with phenylketonuria should be emphasized, in order to prevent associated chronic diseases and to promote health by encouraging continued healthy eating habits and the regular practice of physical exercises.

## RESUMO

**Objetivo:**

Verificar a ocorrência de excesso de peso em crianças e adolescentes com fenilcetonúria e identificar possíveis fatores causais.

**Fontes de dados:**

Revisão sistemática realizada nas bases de dados *Scientific Eletronic Library Online* (SciELO), *Publisher Medline* (PubMed) e Biblioteca Virtual em Saúde (BVS) com os descritores “*Phenylketonurias*”, “*Overweight*”, “*Child*” e “*Adolescent*”. Foram incluídos artigos originais sobre crianças e adolescentes, publicados entre 2008 e 2018 nos idiomas português, inglês ou espanhol.

**Síntese dos dados:**

Foram identificados 16 artigos e, após aplicação dos procedimentos de seleção, 6 estudos foram selecionados para compor a revisão. O excesso de peso em crianças e adolescentes com fenilcetonúria foi evento frequente nos estudos incluídos na presente revisão, variando de 7,8 a 32,6%. Aponta-se o sexo feminino como o grupo mais acometido pelo distúrbio nutricional. O principal fator associado ao excesso de peso na população de interesse na população de interesse foi o consumo calórico elevado aliado à falta de estímulos para a prática de atividades físicas.

**Conclusões:**

O excesso de peso pode ser considerado um desfecho comum em crianças e adolescentes com fenilcetonúria, sendo ocasionado principalmente pelo consumo alimentar inadequado e pelo sedentarismo. Salienta-se a importância da identificação precoce de agravos nutricionais em crianças e adolescentes fenilcetonúricos, a fim de prevenir doenças crônicas associadas e promover a saúde, com incentivo à manutenção de hábitos alimentares saudáveis e à prática regular de exercícios físicos.

## INTRODUCTION

Phenylketonuria (PKU) is a genetic disease, characterized by the total or partial deficiency of the hepatic enzyme phenylalanine hydroxylase, which is responsible for the hydroxylation of phenylalanine (PHE) in tyrosine and results in the accumulation of PHE in the body.^1^ ^,^ ^2^ PKU is characterized as a rare disease, with an incidence in Brazil of approximately one in 16,300 to one in 34,500 live births.^3^

Treatment for the disorder should be instituted early, following a neonatal screening confirming the diagnosis. This is based primarily on the implementation of a restricted diet in foods with high levels of PHE, such as dairy products and all types of meat, fish and eggs.^4^ ^,^ ^5^ ^,^ ^6^ The main objectives of the dietary therapy employed are: to maintain normal growth and development, and to maintain PHE plasma levels within limits to prevent mental retardation, promote the maintenance of plasma tyrosine levels and provide anabolism.^7^

Because the dietary plan of individuals with PKU is limited in protein, including proteins with a high biological value, it contains protein substitutes that have reduced amounts of PHE, which directly interferes with the patient’s needs for dietary growth and development.^4^ ^,^ ^5^

On the other hand, changes in dietary patterns of the general population (increased consumption of high energy density foods and low nutritional value) and the reduction of regular physical activity have led to an overall increase in the incidence of obesity and excess weight in all age groups. According to the World Health Organization (WHO),^8^ in 2016 at least 41 million children were overweight in the world and over 340 million children between the ages of five and 19 were overweight or obese.

The high incidence of overweight in childhood and/or adolescence is worrying, since increased weight in the early years is a predictor of the persistence of this nutritional status in adulthood. Approximately 50% of overweight children at six months of age and 80% obese children at five years of age will remain obese into adulthood.^9^

Regarding the population of individuals diagnosed with PKU, some studies^10^ ^,^ ^12^ ^,^ ^13^ suggest a higher occurrence of patients with PKU being overweight compared to healthy individuals, although the causes are still inconclusive.

In this sense, it is worth noting that the reduction in energy intake from proteins can contribute to a higher consumption of carbohydrate food sources, especially simple and refined carbohydrates (treats, soft drinks, artificial juices etc.) and fats (margarines, vegetable oils). Thus, the proportions of macronutrients in the diet of these individuals generally do not correspond to the constitution of the diet of healthy individuals, which can increase the caloric intake and, consequently, be a predisposing factor for this population group to start to become overweight.^14^

Foreign studies have already detected a higher prevalence of nutritional disorders in individuals with PKU, such as Holm et al.,^15^ who, when comparing the overweight status of 124 children diagnosed with PKU and healthy children, found an increased weight tendency in the group with the disease, especially in females. On the other hand, Allen et al.^16^ , aimed to analyze the energy expenditure of individuals with and without PKU, and not observe the results between the different groups in statistical terms. Their justification was that children with a dysfunctional metabolism from PHE would be predisposed to obesity due to changes in body composition.

In relation to national data on the subject in question, a study conducted in Minas Gerais in 2007, with 125 patients that had PKU and were between two and 12 years old, a prevalence of 8.8% obese and 16.8% overweight was found. In 2009, a study carried out in the same place, with 144 children and adolescents diagnosed with PKU, aged between four and 15 years old, an increase in the rates of previously encountered nutritional disorders was observed, with obesity and being overweight occurring 11.1 and 17.4%, respectively.^14^

Given the above, the present study is justified by the tendency shown in previous studies of overweight individuals diagnosed with PKU and by the repercussions that such changes in nutritional status cause on individuals’ health. Systematically tracing the current literature is quite valuable in the scientific field, because it generates knowledge on a subject that is infrequently studied, such as the nutritional disorders and potential causes of these changes in PKU. By generating such information, it is possible to guide early interventions for health promotion and disease prevention. Thus, the present study aimed to verify, based on the literature, the occurrence of overweight in children and adolescents with PKU, thus evaluating possible causal factors.

## METHOD

### Search Strategy

A systematic review of observational studies was conducted based on the following question: “Do children and adolescents with PKU have a higher occurrence of overweight compared to healthy children and adolescents?” The question was formulated through the PECO strategy, in which each letter of the acronym represents an element of the leading question: P - population, E - exposure, C - control - and O - outcome.^21^ Studies that met the following inclusion criteria were considered to be eligible: original observational studies (cross-sectional, case-control or cohort) developed with humans, published from 2008 to 2018, in Portuguese, English or Spanish, where the results for the population of interest (children and adolescents with PKU) were clearly and separately highlighted. Review articles, experimental articles, articles conducted with adults, and articles that did not present outcomes in separate age groups were excluded from the present study.

Initially, the descriptors to be used were defined according to the Health Sciences Descriptors (DeCS) and the Medical Subject Headings (MeSH): “Phenylketonurias”, “Overweight”, “Child” and “Adolescent”. In order to perform a bibliographic search with a diversity of studies and scientific bases, the following databases were selected for the collection of articles: the Scientific Electronic Library Online (SciELO), the Medline Publisher (PubMed) and the Virtual Health Library (VHL). The entire initial screening of articles was performed in January 2018.

The PubMed search used the following strategy: ((((“phenylketonurias” [MeSH Terms] OR “phenylketonurias” [All Fields]) AND (“overweight” [MeSH Terms] OR “overweight” [All Fields])) AND (“ child “[MeSH Terms] OR” child “[All Fields])) AND (“ adolescent “[MeSH Terms] OR” adolescent “[All Fields] OR” adolescents “[All Fields]) AND (“2008/01/13 “[PDat]: “2018/01/09”[PDat]). In the SciELO and VHL, the search was conducted with the expression: (tw: [Phenylketonuria]) AND (tw: [overweight]) AND (tw: [child]) AND (tw: adolescents).

To minimize a possible loss of publications, a manual search and reference list of articles included in the review were also performed to detect articles that were not retrieved by the database search strategy.

### Study selection

The procedure was independently performed by two researchers using the predefined eligibility criteria for the research.

The screening was subdivided into three parts:

- Title analysis.
- Analysis of the abstracts.
- Reading of the pre-selected articles in full.

The process of identifying eligible articles for the review was done in conjunction with the application of the *Kappa* index^22^ to analyze the agreement between the two researchers. In the end, an excellent agreement was found (κ= 1.0).

The entire description procedure for study identification and selection was based on the Preferred Reporting Items for Systematic Reviews and Meta-Analyzes (PRISMA) guidelines.^23^

We identified 14 articles in the databases searched, 6 indexed in PubMed and 8 in the VHL. No studies were found in SciELO with the descriptors used in the search. Through the manual search, two eligible articles for review were found.

### Quality assessment of the articles

The articles selected for the present study were evaluated for quality through the translated version^24^ of the checklist of the Strengthening the Reporting of Observational Studies in Epidemiology (STROBE) initiative, which features 22 essential items to be included in the observational studies. For quality analysis, each item was scored from 0 to 1. These numbers were then converted to a percentage for better interpretation. Due to the scarcity of papers on the subject, eligible articles were included in the review, regardless of the score obtained.

### Data Extraction

Data were extracted in Microsoft Excel version 2010, in a protocol previously prepared by the researchers, which contained the following information: authors, title, place and year of publication, journal, periodical, study objective, study design, period and place of research, reference population, analyzed/observed variables, instruments used, general population characteristics, sample size, applied statistical analyzes, main results, limitations and quality score.

## RESULTS

**Figure 1:**
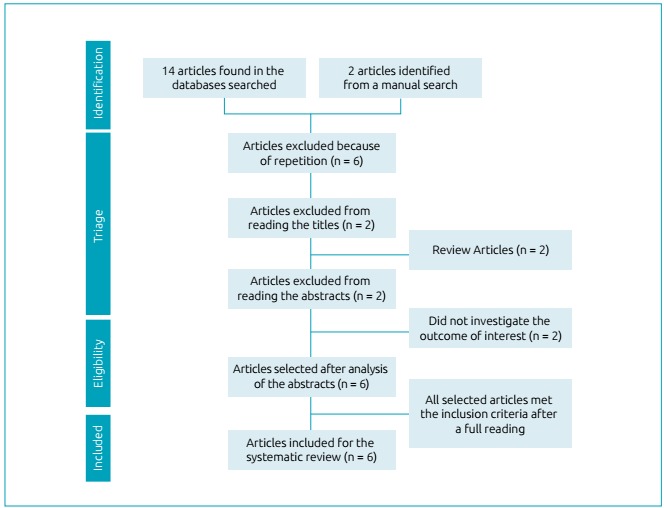
Flowchart of the study identification and selection process for the systematic review of overweight children and adolescents with phenylketonuria.

The steps taken during the study identification and selection process are outlined in Figure 1 and information regarding the general characteristics of the selected articles is present in Table 1. Studies were sorted in descending order according to the score obtained with the STROBE initiative checklist. The median quality score of the articles was 16.2 and it was observed that all had a quality score above 50%. The methodological limitation reported in the base studies in relation to the research design was the retrospective collection.^25^

**Table 1:** Characteristics and quality score of the studies selected for the systematic review.

Reference	Location, year	Design	Population (n)	Population characteristics	Score	%
Aldámiz-Echevarría et al.^28^	Spain, 2013	Retrospective cohort	505	Age range from 1 to 36 years old 46.7% M; 53.3% F	17.1	77.7
Bélanger-Quintana and Martínez-Pardo ^25^	Spain, 2011	Retrospective cohort	160	Age range from 12 months to 28 years old. 46.9% M; 53.1% F	16.7	75.9
Burrage et al.^27^	United States, 2012	Retrospective Study	87	Age range: 2.1 to 20.5 years old 51.7% M; 48.3% F	16.6	75.4
Albersen et al.^10^	Holland, 2010	Cross-sectional	20	Age range: 6 to years old 35% M; 65% F	15.8	71.8
Doulgeraki et al.^29^	Greece, 2014	Cross-sectional	80	Age range: 7 to 10 years old 46.3% M; 53.7% F	15.4	70.0
Rocha et al.^26^	Portugal, 2012	Cross-sectional	89	Age range: 3 to 30 years old 54% M; 46% F	14.6	66.4

M: male; F: female.

Table 2 refers to the main results found in the selected articles. The prevalence of overweight in the population with PKU varied among studies. It was higher in one of the studies^26^, especially in the age group between 10 and 16 years old (39.3% in the PKU versus 12.9% in the similar age control group). One study^27^ identified a higher prevalence of overweight and obese for females with PKU, which was 1.5 to 1.8 times higher than in the reference population, especially in those older than 11 years of age. Albersen et al.^10^ observed statistically significant differences in body fat percentages in girls older than 11 years compared to controls (30.1 ± 5.5% *versus* 21.5 ± 2.2%; p = 0.027).

**Table 2:** Main results of the studies selected for the systematic review.

Reference	Key Results	Indicators analyzed
Aldámiz-Echevarría et al.^28^	Overweight in the PKU group: 9.2% Overweight in the reference population: 17.4% Obesity in the PKU group: 6.5% Obesity in the reference population: 9.8%	Z-scores of weight, height and BMI.
Bélanger-Quintana and Martínez-Pardo^25^	Mean Z-scores for height, weight and BMI were approximately zero for all patients with PKU. There were no statistically significant differences in height, weight and BMI Z scores at any age when compared to sex and age reference values.	Z-scores of weight, height and BMI.
Burrage et al.^27^	Overweight in the PKU group: 12% Overweight in the reference population: 14.8% Obesity in the PKU group: 28% Obesity in the reference population: 16.8%	Z-scores of weight, height and BMI.
Albersen et al.^10^	The mean body fat percentage was significantly higher in patients with PKU compared to healthy controls (25.2 ± 7.3% *versus* 18.4 ± 5.8%; p=0.002).	Weight, height, BMI and body composition assessment.
Doulgeraki et al.^29^	Higher mean BMI Z-scores and body weight of individuals with PKU were observed compared to the control group. There was also a significant increase in fat mass in adolescents with PKU compared to prepubescent patients.	Z scores of weight, height, BMI and a body composition assessment.
Rocha et al.^26^	Overweight in patients with PKU: 32.6% Overweight in the controls: 24.1% Mean body fat (%) in CNS patients and controls were similar: 22.0% (95% CI 14.4-28.9) *versus* 23.1% (95% CI 16.3-28.9); p = 0.581.	Z scores of weight, height, BMI and a body composition assessment.

PKU: phenylketonuria; BMI: body mass index; 95% CI: 95% confidence interval.

Significantly higher numbers were also found compared to the reference population (p <0.001) in the Z-scores for weight and body mass index (BMI) in girls over 13 years old and boys over 18 years old in the group with severe PKU.^25^

It was possible to see that the PKU overweight rates were lower than those of the reference population in almost all age groups considered, with higher percentages above the reference values when the prevalence of obesity was analyzed starting at eight years of age. Compared with the healthy population, the occurrence of obesity in women diagnosed with female PKU was significantly higher in the age group between eight and 18 years old.^28^ A significant increase in body fat mass was also observed in women adolescents with PKU compared to prepubertal patients.^29^

Regarding the possible causal factors for the higher prevalence of overweight in the population with PKU, it was reported that dietary interventions can be a contributing factor, as well as the low incentive to practice physical activities.^25^ ^,^ ^28^ An article^25^ cited changes in body composition (lower percentage of lean mass) as a probable factor influencing excess weight in the individuals analyzed.

Other factors cited as influencers were: choosing high-calorie foods to satisfy appetite, continuing infant formula intake into adulthood^25^ and less supervision in the adolescent age group for formula consumption and meal choice.^27^

## DISCUSSION

The current literature is scarce regarding studies that nutritionally characterize patients with PKU. Some studies consider that individuals with PKU are more vulnerable to weight increase,^11^ ^,^ ^30^ ^,^ ^31^ especially females.^25^ ^.^ ^27^ ^,^ ^32^ According to data published by Gokmen Ozel et al.,^32^ the prevalence of being overweight and obese in the general female population can range from 17 to 35%.

Early identification of changes in the nutritional status of children and adolescents is known to be essential for the prevention of nutritional disorders in adulthood. It also helps prevent the onset of chronic noncommunicable diseases, considering that being overweight is a primary risk factor for the onset of metabolic disorders.^33^ ^,^ ^34^

Kanufre et al.^35^ found higher blood concentrations of triglycerides and basal insulin, a higher total cholesterol/a high density lipoprotein ratio (HDL-c) and index homeostatic model assessment (HOMA-IR). Lower HDL-c concentrations were found in children and adolescents diagnosed with PKU who were overweight compared to eutrophic individuals with PKU. This demonstrates that patients with PKU and excess weight are substantially more susceptible to developing disorders associated with metabolic syndrome.^36^ ^,^ ^37^ ^,^ ^38^ ^,^ ^40^

The present review identified a frequency of overweight ranging from 7.8 to 32.6% in studies including children and adolescents with PKU. This prevalence was lower than that previously found by White and Acosta,^11^ considering that 68.5% of the evaluated children presented the outcome. In a study by McBurnie et al.^30^ significantly higher weight averages were observed between the PKU group at most ages for both sexes (p <0.05) compared to healthy children. Similarly, Acosta et al. demonstrated^31^ high mean values of BMI Z-scores, suggesting important nutritional deviations for overweight children with PKU.

According to the studies analyzed in this review, female subjects with PKU were about 1.5 to 1.8 times more overweight^27^ and had a higher percentage of body fat,^10^ when compared to the group control. According to the authors, this finding may have occurred as a consequence of the girls’ shorter height.^27^ However, the higher number of overweight and obese women is a worldwide trend event, with some exceptions.^41^

On the other hand, a possible causal factor for the higher occurrence of overweight girls is early development.^42^ Females experience growth spurts one to two years before males, at around 9.5 years of age,^43^ and, according to Benedet et al.,^44^ anticipated body and sexual development is closely associated with body fat.

 From a physiological point of view, sex differences in body composition can be explained by the distinct patterns of sex hormone secretion, as well as by the typical differences in lipid metabolism of each sex. These divergences are closely related to reproductive physiology, since body fat, especially located in the lower body, acts as an energy deposit, which enables the female organism to meet the energy costs related to gestational and lactation processes.^45^ Thus, the higher amount of body fat observed in the analyzed studies may reflect the previous preparation of the female organism to perform possible reproductive functions.

In the selected articles,^10^ ^,^ ^25^ ^,^ ^26^ ^,^ ^27^ ^,^ ^28^ ^,^ ^29^ the prevalence of overweight in the PKU group was higher than the reference population when analyzed in older age groups, i.e., in or near adolescence. This higher occurrence may be related to body changes inherent to the intrinsic physiological and endocrine changes of pre-adolescence and adolescence, since in this phase there is an increase of approximately 50% in body weight.^46^

This increase in body weight is related to the changes in the proportions of water, lean mass, fat and bone, which are necessary for the processes of sexual development and growth spurts. Other factors that influence an increase in body weight in adolescence are: a reduction in the number of hours of sleep, eating unstructured meals and, especially, lasting exposure to sedentary leisure activities.^44^

Concerning the practice of physical activities in individuals with PKU, none of the selected studies investigated the association between physical exercise and nutritional status changes, which limited the analysis of the influence of physical inactivity on becoming overweight in the studied group. On the other hand, studies demonstrate that sedentary behavior shown by some individuals can be attributed to the social isolation and anxiety that a strict diet may cause, as well as a lack of organizational skills, which acts as an obstacle preventing individuals from performing routine physical activities.^47^

Overall, although excess weight in children and adolescents was frequent in the studies included in this review, no statistically significant higher numbers were observed when compared to controls of a similar age. Thus, it can be inferred that the condition of being overweight, regardless of the disease analyzed, has been increasing globally in recent years and is mainly associated with a positive energy balance (higher caloric intake associated with physical inactivity), changes in diet composition and also, changes in intestinal microbiota.^41^

In this context, another factor cited^27^ ^,^ ^28^ as a possible cause for the higher occurrence of excess weight and obesity in the PKU group was diet therapy used as a treatment for the metabolic condition in question. In the nutritional therapy of patients with PKU, there was a greater tendency to consume carbohydrate-rich foods in order to complement the individuals’ energy needs, because protein consumption is restricted.

In a study conducted by Burrage et al.,^27^ a higher prevalence of being overweight was observed among individuals categorized as non-dietary compliant, i.e., PJU patients’ poor compliance with the dietary approach was associated with an increased risk of overweight. The authors suggest that the formula with reduced amounts of PHE prescribed in PKU therapy could contribute to the lower incidence of being overweight by inducing satiety and, consequently, decreasing the intake of calorically dense foods. Similarly, Doulgeraki et al.^29^ found a positive correlation between poor adherence to a diet and higher fat mass elevation in patients with PKU.

In their recent work, Jani et al.^48^ observed that the intake of the recommended dietary formulas for PKU was directly proportional to the fat-free mass in children, indicating that adherence to dietary prescription may be associated with favorable outcomes regarding body composition.

Regarding the use of specific dietary formulas in PKU, the results showed the possibility of some patients continuing to consume infant formulas into adulthood. This behavior, which is considered to be inappropriate, may influence weight gain, since such formulas have higher amounts of fat and higher energy content, as they are intended for growing children.^25^ In this regard, the importance of using the age-appropriate formulas prescribed by qualified professionals is emphasized, so that the caloric and nutritional needs of the individual are met without need or excess.

The lower percentage of lean mass was also suggested^25^ as a probable causal factor, as it would induce a lower resting energy expenditure and, consequently, lower total energy expenditure. However, other studies ^16^ ^,^ ^49^ found no evidence of reduced resting energy expenditure in individuals with PKU.

Considering the low prevalence of PKU, the strength of the present study is that it compiles various scientific studies on a subject that is infrequently studied and that is relevant to the field of health. It allows for information to be disseminated, thus enabling early preventive measures to be taken.

Regarding data interpretation from the present review, there may be some limitations to the study, because although the search and screening strategy for the review was clear and systematic, it may not have been able to cover all relevant studies on the subject. The scarcity of studies relating to the theme was also a limiting factor with regard to comparability of results. Additionally, due to the cross-sectional nature of some of the baseline studies, it was not possible to generalize a cause and effect relationship between the presented data.

In short, overweight was a frequent event in children and adolescents with PKU, especially after the age of eight. The selected studies suggest some important factors that may influence the higher occurrence of nutritional deviations in the population of interest, the main ones being: the higher consumption of calorically dense foods due to protein restriction and the lack of stimuli for physical activity due to social withdrawl. Brazilian literature lacks studies on the subject addressed. In view of this, further studies are needed to characterize the nutritional profile of and to assess the risk factors in isolation for metabolic disorders in children and adolescents with PKU, especially nationwide.

Thus, comprehensive nutritional status as well as possible causal factors for endocrinometabolic changes need to be monitored. In their review work, Rocha et al*.* ^50^ proposed the adoption of standardized procedures for PKU weight control assessments to consider dietary, lifestyle, anthropometric and body composition aspects as well as biochemical markers. Such protocols are useful for standardizing the collection of important screening data, as well as for applying early measures to prevent excessive weight gain in the population with PKU.

We hope that this review will stimulate studies on the nutritional profile of children and adolescents with PKU so that the most common nutritional disorders in this group can be identified and help guide the adoption of preventive public health policies. The goal is to prevent nutritional disorders from continuing into adulthood, as well as to reduce risk factors for chronic diseases.
